# A quantitative method for detecting meat contamination based on specific polypeptides

**DOI:** 10.5713/ajas.20.0616

**Published:** 2020-11-25

**Authors:** Chaoyan Feng, Daokun Xu, Zhen Liu, Wenyan Hu, Jun Yang, Chunbao Li

**Affiliations:** 1 Key Laboratory of Meat Processing and Quality Control, MOE; Key Laboratory of Meat Processing, MARA; Jiangsu Collaborative Innovation Center of Meat Production and Processing, Quality and Safety Control; College of Food Science and Technology, Nanjing Agricultural University, 210095, Nanjing, China; 2 Nanjing institute for Food and Drug Supervision and Inspection, 210095, China

**Keywords:** Meat Contamination, Nano-LC-MS/MS, Specific Peptide

## Abstract

**Objective:**

This study was aimed to establish a quantitative detection method for meat contamination based on specific polypeptides.

**Methods:**

Thermally stable peptides with good responses were screened by high resolution liquid chromatography tandem mass spectrometry. Standard curves of specific polypeptide were established by triple quadrupole mass spectrometry. Finally, the adulteration of commercial samples was detected according to the standard curve.

**Results:**

Fifteen thermally stable peptides with good responses were screened. The selected specific peptides can be detected stably in raw meat and deep processed meat with the detection limit up to 1% and have a good linear relationship with the corresponding muscle composition.

**Conclusion:**

This method can be effectively used for quantitative analysis of commercial samples.

## INTRODUCTION

Meat foods are comprised of protein, fat, minerals, vitamins, and other nutrients, which are an indispensable source of human nutrition. In recent decades, meat consumption per capita has been increasing. However, meat safety is a big challenge in some countries or regions. For example, it is quite often illegal to mix low-priced meat, even out-of-date meat with high-priced beef and lamb [1]. Meat contamination is mainly manifested in three forms. Firstly, other animal meats are mixed in a claimed meat. Secondly, meat is replaced by other animal tissues [2]. Thirdly, non-meat components are mixed [3]. Such problems have resulted in the consumers’ serious concerns. Therefore, it is of great significance to develop an accurate, sensitive, and fast method for identifying meat contamination.

In recent years, a lot of qualitative and quantitative techniques have been developed to deal with the problem of meat contamination, including enzyme-linked immunosorbent assay [4–6], near infrared spectroscopy [7,8], electronic tongue [9], electronic nose [10], headspace solid phase microextraction coupled with gas chromatography-mass spectrometry [11], and DNA-based techniques [12–16]. However, these techniques have a relatively low accuracy in meat variety identification and require many samples to establish analytical models. DNA-based methods are not suitable for deeply processed meat products, and it is difficult for the absolute quantification of adulterated components in mixed meat samples.

However, proteomics is an alternative. Montowska and Fornal [17] established triple quadrupole mass spectrometry and multiple reaction monitoring (MRM) model to achieve the absolute quantification of adulterated meat by detecting specific peptide markers in meat (chicken, duck, goose, pork, and beef). Pan et al [18] developed a parallel reaction monitoring mass spectrometry method for detecting trace pork in meat mixtures (chicken, sheep, and beef). Five specific peptides from myosin were selected as external markers. Limit of detection (LOD) in mixed meat was up to 0.5%. Prandi et al [19] established a liquid chromatography mass spectrometry (LC-MS) method with good linearity for identification and quantification to discriminate eight different meat products. Compared with other methods, mass spectroscopy can identify specific peptides from different animal samples, in mixed meat samples. Peptides that are tolerant to cooking, baking, drying and sterilization are more suitable for the identification of adulterated meat in processed meat products [20]. However, few data are available on such a technique.

In this study, we established a method for efficiently screening specific polypeptides. In addition, thermally stable polypeptides were applied to detect commercial samples and the detection limit was up to 1%.

## MATERIALS AND METHODS

### Screening of specific peptides

Fresh chicken, duck, pork, beef, and lamb samples were obtained, and visible fat and connective tissue were removed. Half of meats from each species were cooked in an 80°C water bath for 60 min. Then the raw and cooked samples were homogenized in 20 mL 2% sodium dodecyl sulfate phosphate buffered saline buffer (0.1 mol/L, pH 8.0). The homogenization conditions were 9,600 rpm for 30 s, and 13,400 rpm for 30 s, and the interval between two bursts was 30 s. Then, samples were centrifuged at 4°C at 4,000 g for 10 min. The supernatant was collected and filtered through gauze. Protein content in the supernatant was quantified by a commercial BCA kit (Thermo Fisher Scientific, San Jose, CA, USA) according to the manufacturer’s instructions.

Protein samples (200 μg) were transferred into 10 kDa ultrafiltration tubes and centrifuged at 14,000 g for 15 min. Then sample buffer (200 μL 8 M urea, 50 mM Tris-HCl pH8.0) was added and centrifuged again (14,000 g, 15 min). The same sample buffer (200 μL 8 M urea, 50 mM Tris – HCl, pH8.0) containing 5 μL 1 M dithiothreitol (DTT) was added to reduce disulfide bonds. The mixture was heated at 60°C for 60 min, and then centrifuged at 14,000 g for 15 min. The sample buffer (200 μL 8 M urea, 50 mM Tris-HCl, pH 8.0) containing 20 μL 0.5 M iodoacetamide was mixed and kept in dark for 45 min, and centrifuged again (14,000 g, 15 min). Then 200 μL 50 mM NH_4_HCO_3_ (pH7.8) was added and centrifuged at 14,000 g for 15 min, and the step was repeated once. The bottom tubes were replaced by new ones, and 200 μL 50 mm NH_4_HCO_3_ (pH7.8) and 4 μg trypsin were added. Then the samples were incubated at 37°C for 16 h. After the incubation, 50 μL 50 mM NH_4_HCO_3_ (pH7.8) was added and centrifuged at 14,000 g for 25 min. The filtrate was retained and stored at −20°C.

The filtrate was loaded onto the extraction head of Monolithic spin columns (MonoSpin, GL Sciences, Tokyo, Japan) for desalination as described previously [21]. Briefly, the column was rinsed stepwise by 100 μL 60% acetonitrile (ACN) solution (0.2% formic acid), 100 μL 0.2% formic acid, 100 μL of the former filtrate and 300 μL 0.2% formic acid. At each step, the samples were centrifuged at 5,000 g for 2 min and the filtrate was discarded. Then the bottom tubes were replaced by new ones and 100 μL 60% ACN solution in 0.2% formic acid buffer was added to elute the samples by centrifuging at 5,000 g for 2 min. The desalted samples (the filtrate) were collected, and the concentration was determined by a Nanodrop spectrophotometer (Thermo Fisher Scientific, USA).

Peptide products were separated by reversed phase high-performance liquid chromatography. The nanoliter ion source of the LTQ-orbitrap mass spectrometer (Thermo Fisher Scientific, USA) was used to analyze and detect peptide products. The peptide mixture was loaded into a C_18_ chromatographic column (2 cm×200 μm, particle size 5 μm) by an automatic sampler. Samples were separated by a C_18_ chromatographic column (75 μm×100 mm, particle size 3 μm). Mobile phase A (0.1% formic acid solution) and mobile phase B (0.1% formic acid, 84% acetonitrile aqueous solution) were used. Gradient elution conditions were 0 to 12 min (97% A, 3% B), 12 to 100 min (72% A, 28% B), 100 to 120 min (45% A, 55% B), 122 to 144 min (2% A, 98% B), 144 to 160 min (97% A, 3% B), and the flow rate was 300 nL /min. The separated peptides were scanned on the LTQ Orbitrap XL platform. The collision-induced deionization normalized collision energy was set at 35. Fragments were detected in the linear ion trap at normal resolution. The locking mass was set at 445.120020, and the total scan ranged from 300 to 1,800 m/z within 160 min. Proteome Discover −1.4 (Thermo Fisher Scientific, Palo Alto, CA, USA) was used to match peptide level 2 mass spectrometry data (http://www.uniprot.org/) against pork (*Sus scrofa*), beef (*Bos Taurus*), chicken (*Gallus Gallus*), lamb (*Ovis Aries*), and duck (*Anas platyrhynchos*) databases. The search parameter was set as follows: the parent ion concentration tolerance was 10 ppm, the Oxidation of Met was set as variable modification, and the number of allowed missing cut sites was 2.

### Testing commercial samples

Triple quadrupole series high performance liquid chromatography and mass spectrometry instrument has a lower resolution, but it is quantitative accurate and has a higher popularity. And thus, the instrument was applied to further screen specific peptides. Triple quadrupole series high performance liquid chromatography and mass spectrometry (Agilent 6495, Agilent Technologies, Santa Clara, CA, USA) was used for the specificity verification, thermal stability verification, standard curve drawing of specific peptides and test of commercial samples. Raw and cooked samples (80°C, 1 h) (chicken, duck, pork, beef, and lamb) were used for thermal stability verification. Two meat species were mixed at ratios of 0 to 100, 20 to 80, 40 to 60, 60 to 40, 80 to 20 and 100 to 0 for standard curve drawing of specific peptides. Three combining regimes were applied by mixing pork with beef, or duck with beef, or chicken with lamb. The specific ingredients were listed in Table 1; Two meat species were mixed at a ratio of 1 to 99 for the specificity verification. Two combining regimes were applied by mixing 1% chicken with lamb and mixing 1% duck with beef. The meat samples were treated stepwise with 70% ethanol, 100% ethanol, 90% methanol and deionized water, for 30 s each time. Then, 1 g meat samples were added to 5 mL protein extraction buffer (7 M urea, 2 M thiourea, 50 mM DTT, 4% 3-[(3-Cholamidopropyl) dimethylammonio]-1-propane) and homogenized on ice. The homogenate was centrifuged at 12,000 g for 10 min at 4°C. Four volumes of acetone was added and vortexed completely. The samples were centrifuged at 12,000 rpm 4°C for 10 min and the pellets were dried. The dried samples were dissolved in 1 mL 1 M urea in 100 mM NH_4_HCO_3_. The protein concentration was determined with a commercial BCA kit (Solarbio, Beijing, China). The protein samples (100 μg) were mixed with 50 μL 100 mM NH_4_HCO_3_ containing 10 mM DTT and kept at 60°C for 30 min. Then 50 μL 100 mM NH_4_HCO_3_ containing 55 mM iodoacetic acid was added and placed in dark for 20 min at 25°C. Then the samples were incubated with 10 μL trypsin (0.1 mg/mL in 25 mM NH_4_HCO_3_) for 16 h at 37°C. The digestion was stopped by adding10 μL formic acid. A prepared C_18_ column was loaded and activated by adding 5 mL methanol and being followed by 5 mL 1% formic acid. The samples were loaded onto the columns and washed with 5 mL 5% methanol (containing 1% formic acid) and eluted with 5 mL 90% acetonitrile (containing 0.1% formic acid). Finally, 25 μL dimethyl sulfoxide was added and blown dry with nitrogen and finally dissolved in 1 mL 3% acetonitrile in 0.1% formic acid. The peptides (20 μL) were separated in Agilent Poroshell 120 EC-C18 column (particle size: 2.7 μm, 150×3.0 mm) and the flow rate was set at 0.3 mL/min. Column temperature was set at 35°C. Mobile phases A (acetonitrile containing 0.1% formic acid) and B (0.1% formic acid) were used. The procedures for gradient elution are listed in Table 2. Mass spectrometry was performed with an electrospray ionization (ESI) ion source (+ion mode). Nitrogen was applied at gas temperature of 250°C and sheath temperature of 350°C with a flow rate of 12 L/min and atomization pressure of 25 psi. Capillary voltage was set at 4,000 V and MRM scanning mode was applied.

### Statistical analyses

The measured data were analyzed by one-way analysis of variance and means were compared by Duncan’s multiple comparison under SAS program (version 8.1.2, 2009).

## RESULTS AND DISCUSSION

### Efficiency of sample pretreatment

**Figure 1:**
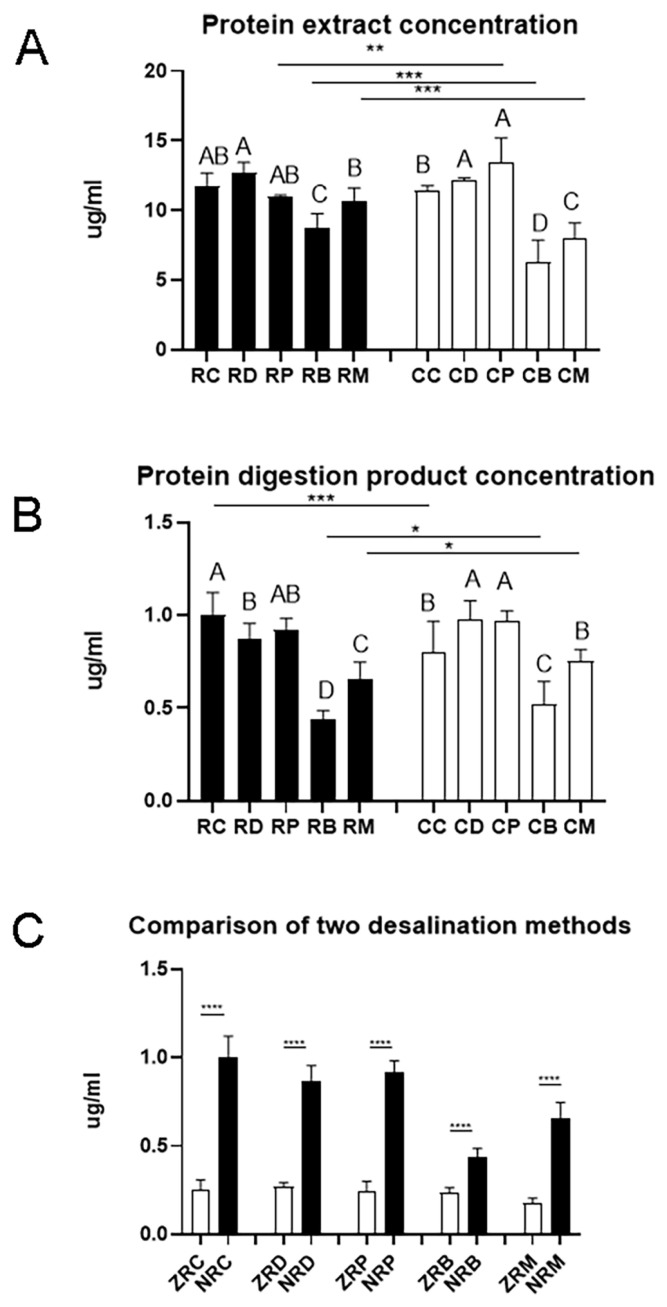
The efficiency of protein extraction varied with the source of samples and the state of meat. (A) The yields of protein extraction were different among meat species and between raw and cook meats. (B) Protein digestion also showed differences among meat species and between raw and cooked meats. (C) Monospin column had a better desalting result. RC, RD, RP, RB and RM, raw chicken, duck, pork, beef and mutton; CC, CD, CP, CB, and CM, cooked chicken, duck, pork, beef and mutton; ZRC, ZRD, ZRP, ZRB and ZRM, the digestion products of raw chicken, duck, pork, beef and mutton passing through the Ziptip column; NRC, RD, RP, RB and RM, the digested products of raw chicken, duck, pork, beef and mutton passing through the Monospin column. A, B, C indicate significant differences among meat species. Data are shown as means and standard deviations.

The matrix in meat samples is complex. In targeted proteomics, efficient protein extraction is one of the most critical steps for analyzing processed meat samples [22]. In both raw meat and cooked meat samples, the protein concentration of beef and lamb was significantly lower than that of duck, which could be due to the presence of more non-protein components in the beef samples (Figure 1A). After cooking, the protein concentration significantly increased for pork samples (p<0.01) but decreased for beef and lamb samples (p< 0.001). During the cooking process of beef and lamb, some soluble proteins may undergo denaturation and become insoluble [23], and thus the protein concentration is reduced.

After trypsin digestion, the peptide concentration differed greatly with meat species and cooking even if the initial protein contents were the same (Figure 1B). The peptide concentration was the lowest for beef digestion samples (p<0.05). The peptide concentration in cooked chicken digestion samples was significantly lower than that in raw chicken digestion samples (p<0.001). However, the peptide concentration in digestion samples of cooked beef and cooked lamb was significantly higher than their counterparts of raw meat (p<0.001). This indicates that proteins in chicken showed a different vulnerability to heating and subsequent trypsin digestion from proteins in beef and lamb.

Desalting is a critical step for mass spectrometry. Solid phase extraction (SPE) is one of the most common protein desalination methods. Monolithic spin column, a relatively new SPE product that has not been widely used, can enhance sample preparation speed and accommodate small sample volumes [24]. The Ziptip C_18_ column is a traditional method with micropipette-tip SPE. Palmblad and Vogel [25] found that the ZipTip C_18_ column bound and recovered more sample than the other two types of C_18_ tips from identical samples using the same loading and elution conditions. Robustness, reproducibility, sensitivity, and economic parameters encompassing time and costs must be addressed along with the selection of proper SPE product [26]. In the present study, yields of polypeptide products were compared between a traditional Ziptip column and a new monolithic SPE column (Monospin C_18_ column). Compared with the ziptip column, the Monospin C_18_ column was more efficient removing salt and the peptide yield increased greatly (Figure 1C). In practice, the operation of the ziptip column is complicated, and only 10 to 20 samples can be prepared in one hour. However, for the Monospin C_18_ column, it would be possible to prepare up to more than 100 samples per hour with a sample preparation time of 15 to 20 min.

### Selection of specific peptides

Pork, beef, lamb, chicken, and duck have their own specific peptides, which have specific amino acid sequences and are present at high levels in muscle tissues but absent or low in non-muscle tissues. In addition, these peptides are generally of high thermal stability. The traditional method to screen specific peptides was to match the sequences of a specific protein from one meat species to another species in National Center for Biotechnology Information or Uniprot database. The different sequences could be considered as species-specific peptides. However, this screening method does not work well because the screened peptides do not always respond well in an actual test [27–28]. Therefore, finding novel species-specific peptide biomarkers requires comprehensive and detailed observation of meat protein homologies [29]. In the present study, species-specific peptides were screened by trypsin-digestion and high resolution liquid chromatography combined with tandem mass spectrometry.

**Figure 2:**
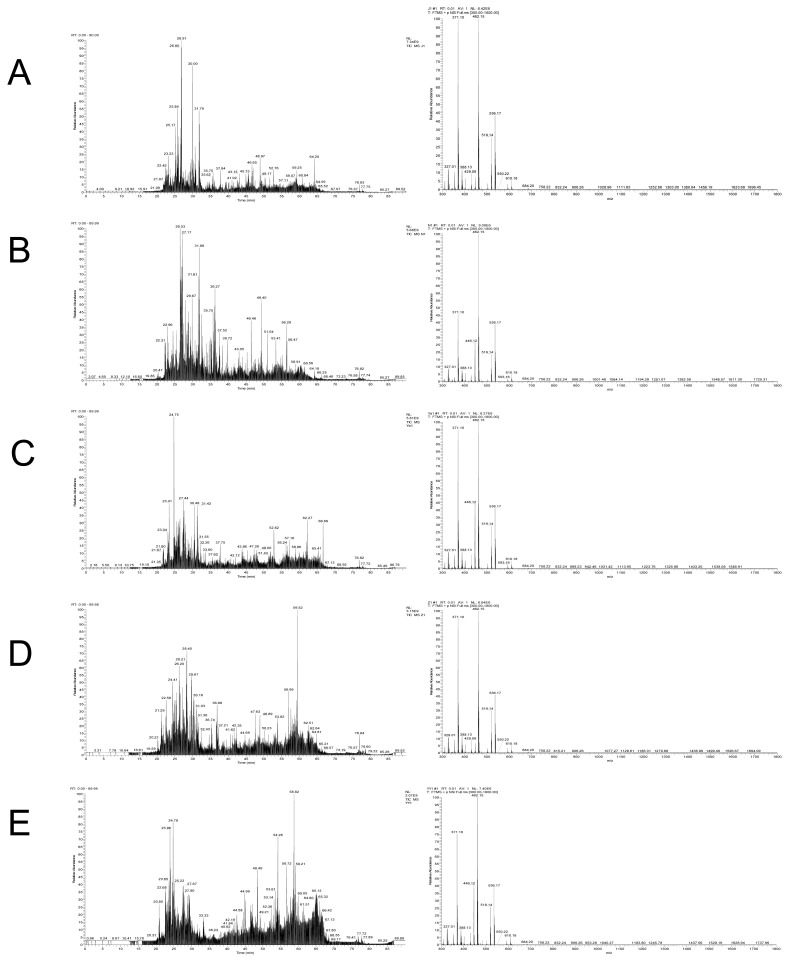
Diagrams of total ion flow and mass spectrometry from digested products of chicken (A), beef (B), duck (C), pork (D), and lamb (E).

A total of 794 peptides were identified from the digestion products of raw chicken, corresponding to 1,007, 1,052, 1,571, and 1,181 for raw beef, duck, lamb, and pork, respectively (Figure 2). In digestion products of cooked meat, the numbers of identified peptides increased greatly. A total of 1,218, 1,472, 1,194, 1,662, and 1,170 peptides were identified from cooked chicken, beef, duck, lamb, and pork, respectively. A previous study showed a decrease in the number of peptides in cooked samples and a decrease in protein sequence coverage [30]. However, a different phenomenon was found in the present study. In general, heat treatment improves meat degradation potential, which may cause proteins to be broken down more thoroughly into peptides [31].

After careful comparison, 146 peptides were selected specific for chicken, and 218 specific for duck, 181 specific for pork, 93 specific for beef and 111 specific for lamb as well (Supplementary File 1). Notably, these species-specific peptides are not always applicable considering the following factors: high abundance, good signal to noise ratio at low concentrations, high specificity, no missing cleavages, and trypsin specific cleavage sites at both ends [19]. For example, considerable part of peptides can be detected by the LTQ-orbitrap mass spectrometer but cannot be detected by triple quadrupole mass spectrometry. And thus, further screening and verification were performed.

Ten specific peptides with the highest responses were selected for each species, and the retention time, sub-ion matching degree and corresponding strength of each group of specific ion pairs were investigated. Peptides were selected that were stable in the measured samples. The finally selected specific peptides are shown in Table 3. These peptides are composed of 9 to 17 amino acids. The size range of precursor ion is 513 to 876 kDa. Among them, the chicken-specific peptide LDVPISGEPAPTVTWK has been reported [17,18]. In addition, other peptides have not been reported and may provide alternative biomarkers for the relevant methods.

### Reliability of species-specific peptides

The thermal stability of selected species-specific peptides was tested. The application of them to the actual samples from raw and cooked meat was evaluated.

**Figure 3:**
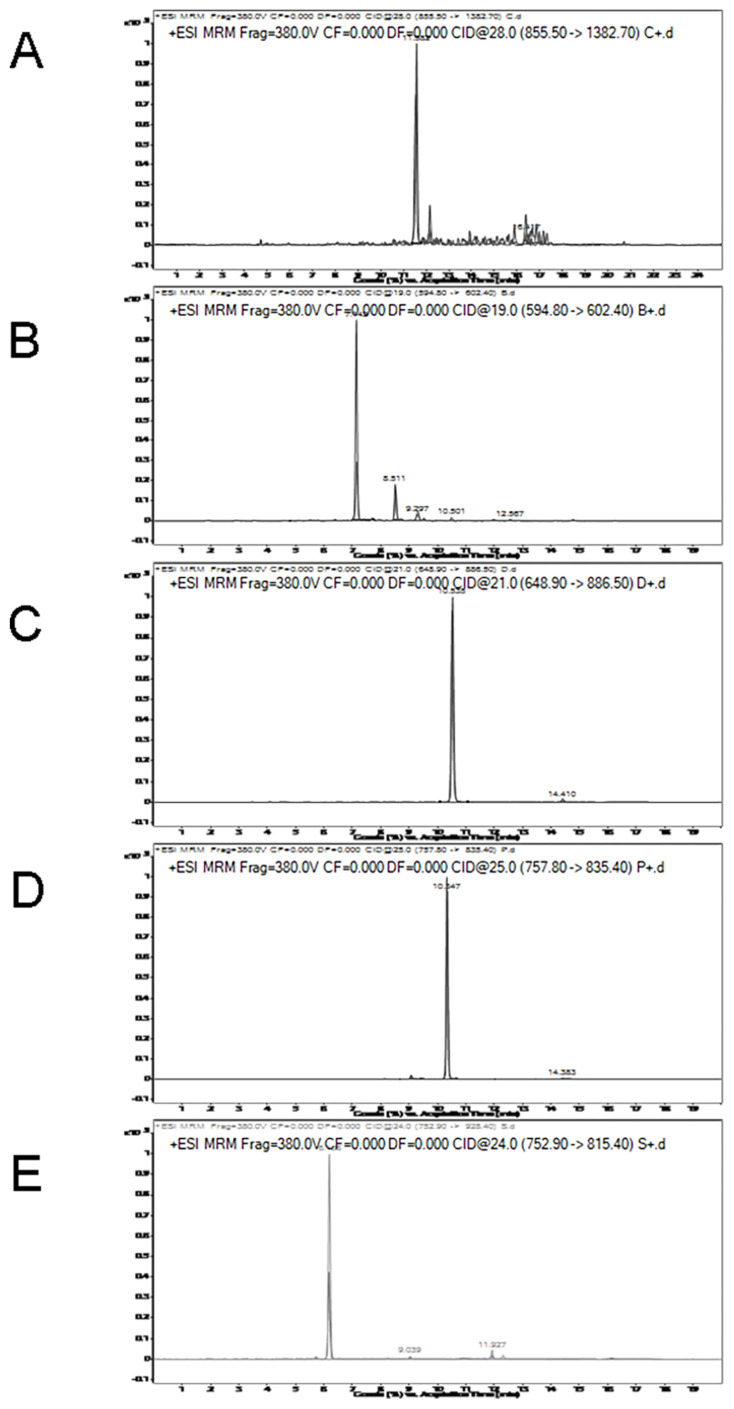
Multiple reaction monitoring (MRM) transition intensities and retention times of specific peptides varied among chicken (A), beef (B), duck (C), pork (D), and lamb (E).

MRM transition intensities versus retention time for chicken, beef, duck, pork, and lamb specific peptides are shown in Figure 3. As shown in Figure 3C and D, the retention time of the duck-specific peptide (LAILENANVLAR) is 10.535, which is close to the pork-specific peptide (DQGSYEDFVEGLR) of 10.347. MRM transition intensities of these two peptides were not significantly different between raw meat and cooked meat. The retention times for other specific peptides are shown in Table 3. MRM transition intensities of the chicken, lamb and beef-species peptides, that is, LDVPISGEPAPTVTWK, NLVHIITHGEEKD, and TLEDQVNELK were reduced by nearly 30%, 60%, and 70% from raw meat to cooked meat, respectively. Although the responses of some specific peptides decreased, they are still suitable for quantitative testing.

**Figure 4:**
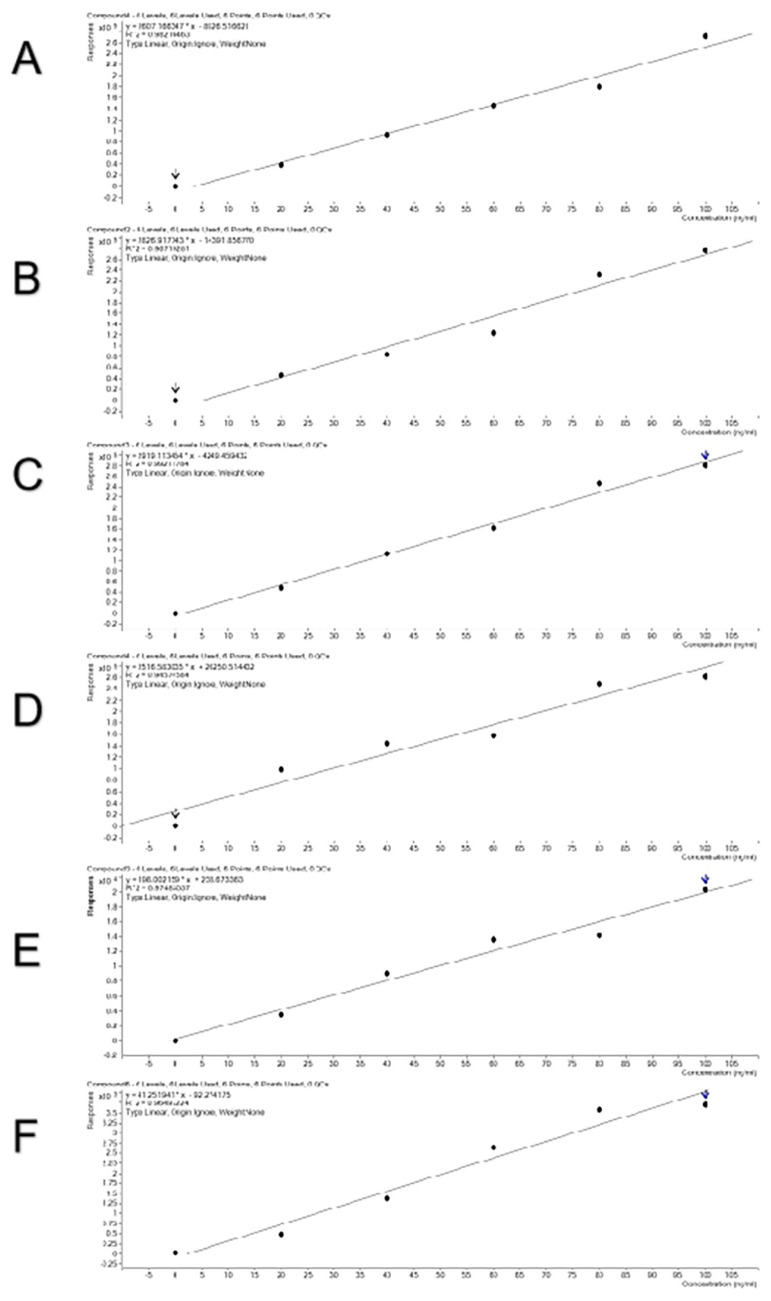
The standard curves of the mixed model for adding beef to pork (A), pork to beef (B), duck to beef (C), beef to duck (D), lamb to chicken (E), and chicken to lamb (F).

To verify a good linear relationship between MS intensities of these peptides and their real contents in meat, combinations of two meat species samples were done in a certain proportion and standard curves were obtained (Figure 4). The curves were linear and the R^2^ values reached 0.946 to 0.992, indicating that the selected species-specific peptides are suitable for quantitative detection. All the standard curves show relatively high reliability with high R^2^ values. Three combinations of pork and beef, duck and beef, and chicken and lamb did not differ in R^2^ values. The mixing order did not affect R^2^ values, either.

**Figure 5:**
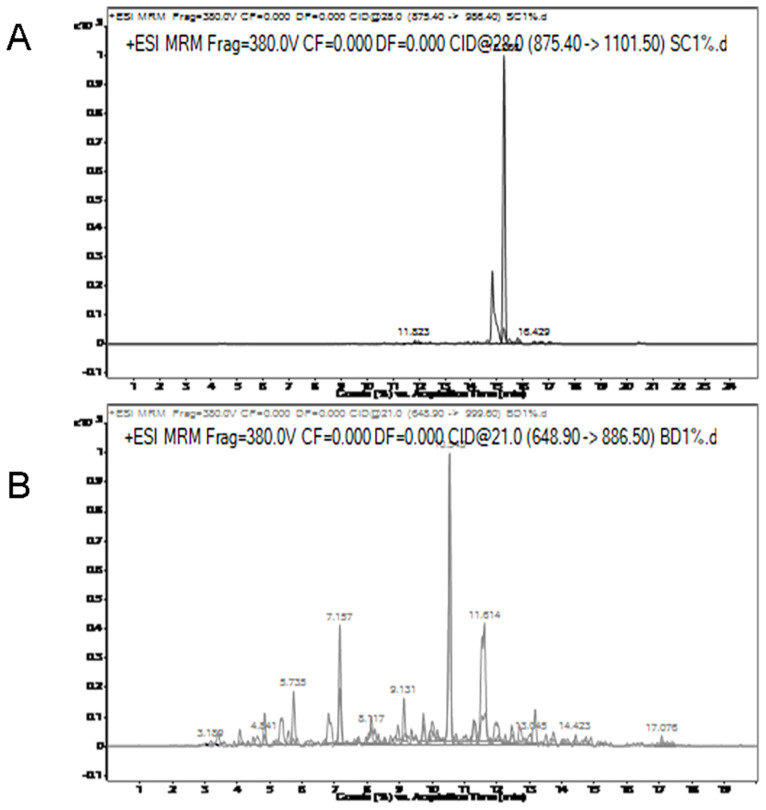
Multiple reaction monitoring (MRM) transition intensities and retention times for 1% chicken in lamb (A) and 1% duck in beef (B).

To test the LOD of the method, 1% chicken meat was mixed with 99% lamb, and 1% duck meat was mixed with 99% beef. The results indicated that chicken-specific peptides and duck-specific peptides can be detected in beef and lamb respectively (Figure 5). This indicates that the method has a good detection limit. In fact, the percentage of contamination is much higher than 1%.

**Figure 6:**
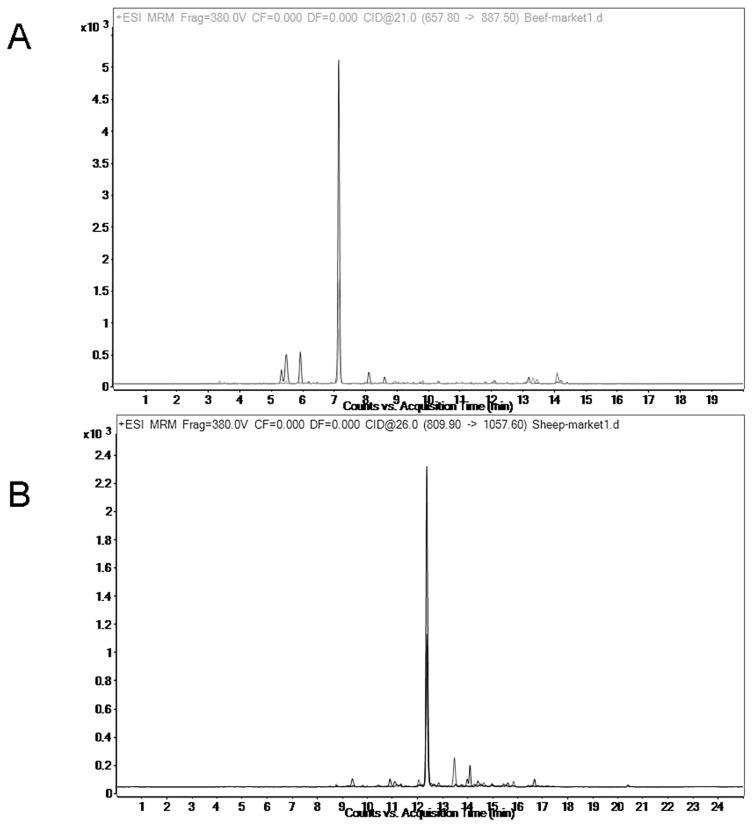
Multiple reaction monitoring transition intensities and retention times for commercial samples. Pork components was found in beef (A) and chicken components was found in lamb (B).

In addition, several samples obtained from the market were by this method. As shown in Figure 6, pork components (VNVDEVGGEALGR) were found in beef and chicken components (IGDEFVADLDQLQR) were found in lamb. After a single sample preparation, the components of the sample can be determined with a variety of specific peptides. In addition, the specific peptides of the same species can be used at the same time for further verification of the test result.

In summary, we proposed a LC-MS/MS-based method to detect meat contamination using five meat species, i.e., beef, pork, lamb, chicken, and duck. Efficiency of protein extraction showed a great difference among meat species with lower yields for beef and lamb, and between raw and cooked meat as well. The Monolithic spin column is better to desalt than the Ziptip column. Species-specific peptides were screened by nano LC-LTQ-Orbitrap XL and ESI mass spectrometry. Finally, 15 peptides were selected for identifying meat contamination by triple quadrupole series high performance liquid chromatography and mass spectrometry. The LOD reached 1%. The proposed method is promising for detecting meat contamination.

## Figures and Tables

**Table 1:** The specific ingredients for standard curve drawing of specific peptides

Group	The sample components					
1	100% pork	20% beef+80% pork	40% beef+60% pork	60% beef+40% pork	80% beef+20% pork	100% beef
2	100% beef	20% pork+80% beef	40% pork+60% beef	60% pork+40% beef	80% pork+20% beef	100% pork
3	100% beef	20% duck+80% beef	40% duck+60% beef	60% duck+40% beef	80% duck+20% beef	100% duck
4	100% duck	20% beef+80% duck	40% beef+60% duck	60% beef+40% duck	80% beef+20% duck	100% beef
5	100% chicken	20% lamb+80% chicken	40% lamb+60% chicken	60% lamb+40% chicken	80% lamb+20% chicken	100% lamb
6	100% lamb	20% chicken+80% lamb	40% chicken+60% lamb	60% chicken+40% lamb	80% chicken+20% lamb	100% chicken

**Table 2:** Procedures for gradient elution

Time/min	A/%	B/%
0.00	10	90
15.00	40	60
15.01	100	0
18.00	100	0
18.01	10	90
20.00	10	90

**Table 3:** Selected species-specific peptides

Species	Compound name	Precursor Ion	Product Ion	RT	CE	Serial number
Beef	TLEDQVNELK	594.808967	845.436323	7.142	19.4	SEQ ID NO.1
Beef	GLSDSVSIGPVTVK	679.879924	899.556044	9.465	22.1	SEQ ID NO.2
Beef	FLEELLTTQC	762.871773	1006.49861	10.549	24.6	SEQ ID NO.3
Chicken	LVSWYDNEFGYSNR	875.397001	1264.52291	14.072	28.1	SEQ ID NO.4
Chicken	IGDEFVADLDQLQR	809.907201	1057.56365	12.472	26.1	SEQ ID NO.5
Chicken	LDVPISGEPAPTVTWK	855.45907	1382.73144	11.566	27.5	SEQ ID NO.6
Chicken	ECQTLVSDVDYR	742.83793	966.489087	9.529	24	SEQ ID NO.7
Duck	VVFDDSFDR	550.256371	901.368637	8.38	18.1	SEQ ID NO.8
Duck	IVESLQSSLDAEIR	780.417402	1018.51636	9.645	25.2	SEQ ID NO.9
Duck	LAILENANVLAR	648.885344	999.558169	10.535	21.1	SEQ ID NO.10
Pork	VNVDEVGGEALGR	657.836048	887.458121	7.124	21.4	SEQ ID NO.11
Pork	DQGSYEDFVEGLR	757.841527	1127.53677	10.346	24.5	SEQ ID NO.12
Lamb	SPPNPENIAPGYSGPLK	869.443951	1342.70014	7.954	28	SEQ ID NO.13
Lamb	HVLTTLGER	513.290548	789.446494	5.044	16.9	SEQ ID NO.14
Lamb	NLVHIITHGEEKD	752.891354	1041.52112	6.188	24.3	SEQ ID NO.15
