# Supplementary material for: A quantitative method for detecting meat contamination based on specific polypeptides
Source: Anim Biosci. 2020 Nov 25;34(9):1532–43. doi: 10.5713/ajas.20.0616 (PMC8495334; doi:10.5713/ajas.20.0616)
Supplement: Supplementary file 1 [file ajas-20-0616-suppl.pdf]

A quantitative method for detecting meat contamination based on specific polypeptides

Chaoyan Feng[1], Daokun Xu[2]， Zhen Liu[2]， Wenyan Hu[2]， Jun Yang[2]， Chunbao Li*[1]

[1]Key Laboratory of Meat Processing and Quality Control, MOE; Key Laboratory of Meat Processing, MARA; Jiangsu Collaborative Innovation Center of Meat Production and Processing, Quality and Safety Control; College of Food Science and Technology, Nanjing Agricultural University, 210095, Nanjing, China

[2]Nanjing institute for Food and Drug Supervision and Inspection

Corresponding author:

Prof. Dr. Chunbao Li

E-mail: chunbao.li@njau.edu.cn

Tel/Fax: 86 25 84395679

Table S1 Specific peptides for beef, pork, lamb, chicken, duck

Annotated Sequence	Theo. MH+ [Da]
**Beef**	
[R].ALPAAAIEGPAYNR.[A]	1413.748489
[R].TPVDPPGKPEVIDVSK.[S]	1677.905777
[R].VYSENVCGLSDLPGVSK.[N]	1823.88439
[K].VDKGDSGQYTCYAVNEVGK.[D]	2089.94951
[K].VGLGEATAVPGTVKPEDK.[L]	1767.948705
[K].GDSGQYTCYAVNEVGK.[D]	1747.75919
[R].NAQLDDQANYNVSLTNHR.[G]	2072.974419
[K].AIGIVATTGVGTEIGK.[I]	1486.847534
[R].YSIQNTDSSSILTIPQVTR.[H]	2123.097888
[K].LVNELTEFAK.[T]	1163.630665
[R].VAAVSSAGAGPPAVLER.[L]	1551.848931
[R].YGISGSDQTLTIR.[Q]	1410.722334
[R].KVPQVSTPTLVEVSR.[S]	1639.937746
[R].TSDYEQSETSRPALAQPVPEKPVER.[K]	2814.390441
[K].TLAFLFSGTPTGDSEASGGTK.[K]	2043.986941
[K].INLDGLDADNTVTVIAGNK.[L]	1943.00801
[K].EFTPVLQADFQK.[V]	1422.726357
[R].VLQELVNDGADDRPAGTR.[S]	1925.967543
[R].DPCDPPGRPEPIIVTR.[N]	1818.916693
[K].TVTNLACVVTGLQQGK.[T]	1688.899981
[K].NCAVADESVYGFK.[L]	1459.652206
[K].GLSDSVSIGPVTVK.[E]	1358.752571
[K].VTNPVGEDVASILLK.[V]	1554.873749
[R].AEWEEVTNSAVER.[R]	1519.702327
[K].TLALLFSGPASGEAEGGPK.[K]	1801.933055
[K].AGEDVHALVPFK.[G]	1282.679013

[R].KDVASTEWSPLSTTSK.[K]	1736.87012
[R].YIIEAVNVCGR.[A]	1293.661983
[R].ILDQYLYK.[E]	1055.577173
[K].AVNAAGVSKPSATVGPVIVK.[D]	1865.085473
[K].EENEVLAPAPAPPPEEPNKEK.[E]	2285.129582
[R].IPADVDPLAITSSLSSDGVLTVNGPR.[K]	2594.367186
[K].FLEELLTTQCDR.[F]	1524.73627
[R].IEDEQVLGSQLQK.[K]	1486.774763
[K].EEVILQCEISK.[A]	1347.682443
[K].LSDTSTLVGDAVELR.[A]	1575.822442
[R].NAAGVFSEPSESTGAITAR.[D]	1864.903545
[K].TAQDPISPPGPPVPR.[V]	1528.811818
[K].LPGDQWIR.[C]	984.5261409
[R].LNLEAINYLSAGGDFK.[I]	1724.885376
[K].EPPVLIVTPLEDQQVFVGDR.[V]	2251.196874
[R].ILTVEGLTEGSLYEFK.[I]	1798.947308
[K].VEFPQDQLTTLTGR.[I]	1604.827861
[K].DVASTEWSPLSTTSK.[K]	1608.775157
[K].IQGTLEDQIISANPLLEAFGNAK.[T]	2442.28748
[K].LNAYNLSDNIYK.[H]	1427.71652
[K].LKPDPNTLCDEFK.[A]	1576.76757
[R].SAIYPTSAEHVGAALK.[S]	1614.848597
[R].EAPVDLLSQIGSSVR.[G]	1570.843511
[R].VVVAGYAAALNCAVR.[G]	1533.820608
[K].FVAADVGEYQCR.[V]	1414.641975
[K].DGQELQLVDR.[I]	1172.590591
[R].SEWSDLLSDLQK.[SX]	1420.69545
[K].DQTVIQNTDGNNNEAWAK.[N]	2017.920986
[K].TTGLDEGLEYEFR.[V]	1529.711829

[R].ISFEELLK.[V]	978.5506243
[R].TLEDQVNELK.[S]	1188.610658
[K].SIFTSVPDTPDLTR.[A]	1548.790413
[R].DIASDYLYK.[T]	1087.530617
[K].ELVIPPEVDLSEIPGAQVAVR.[I]	2231.228174
[R].ETTSTTWHVVSQAVAR.[T]	1772.892587
[K].DFCDSCAFNIDVEAPR.[Q]	1915.794924
[R].SGQYDGLVELATICALCNDSSLDFNETK.[G]	3120.413621
[K].AAAAPAPAPAPPPAPEPSKEPEFDPSK.[I]	2637.319508
[R].EPVPVPIPAALLR.[E]	1371.835848
[R].ELLLPVLIK.[E]	1037.696895
[R].AAAEGGSSSVFSMFDQTQIQEFK.[E]	2465.12893
[K].YDGGSQVTNYIVLK.[R]	1556.795499
[R].GKPIPDIVWSK.[D]	1239.709584
[K].LNQNLNDVLISLEK.[Q]	1612.890462
[R].ILESGPFVSCVK.[K]	1335.697699
[K].DLQTEASLDTVLCYDPAAAK.[W]	2181.03799
[R].VAAENAAGVGEPSEPSVFSR.[A]	1973.956309
[R].AVVEGFQPISVVWLK.[D]	1671.946854
[R].AGTPFVPELTSGLELLR.[L]	1799.990176
[R].APVPASELLASGILSSSQFEQLK.[D]	2372.270767
[R].VSGSAPISVGWFQDGNEIVSGPK.[C]	2331.161551
[K].DPASGICTLLYDSAPSGR.[F]	1879.885453
[K].EYLPIAGLAEFCK.[A]	1510.761028
[K].IDAAEEEKYDMEIR.[V]	1711.784342
[RK].DILSQLGIK.[K]	986.5880724
[R].AALGESTLGETTLGETSLGDTTLGR.[T]	2450.225667
[R].YLLDEYSAK.[K]	1101.546267
[R].LYQGAGGPGAGGFGAQGPK.[G]	1689.834344

[K].GTFAALSELHCDK.[L]	1448.68384
[K].DLADEVALVDVMEDK.[L]	1661.793844
[K].HGEIESNVNLQVDER.[K]	1738.835466
[K].ALDGSALYTGSALDFVR.[N]	1755.89119
[R].STVSLIWSAPVYDGGSK.[V]	1766.895941
[R].VSAENAAGVGEPSPATLYYK.[A]	2023.997111
[K].IIKPPPVEPPPTPIAAPVTVPVVGK.[K]	2513.510544
[K].SDETDWQTAIQNLR.[G]	1676.787453
[K].YDGGSDIINYVLESR.[L]	1700.812605
**Pork**	
[R].EVTLDDISQIK.[A]	1260.668173
[K].VLDSPGPPAGVTVR.[D]	1364.75324
[K].VDFPQDQLSTLTGR.[I]	1576.796561
[K].EAVLGLWGK.[V]	972.551293
[R].ISSSNPYSTVTVDDIR.[A]	1753.860284
[K].AVNAAGVSRPSATVGPVTVK.[D]	1881.055235
[K].TLAFLFSGAQTGEAEAGGTK.[K]	1955.970897
[R].ELQAFENIEDEIK.[L]	1577.769343
[K].GQTVQQVYNAVGALAK.[A]	1646.886045
[K].VGLGEATSVPGTVKPEDK.[L]	1783.943619
[K].DVYVPDDKEEFVK.[A]	1582.76353
[R].VCAENENGEGTPSEITAVAK.[D]	2075.954989
[K].LPADTEVVCAPPTAYIDFAR.[Q]	2206.084881
[K].DQGSYEDFVEGLR.[V]	1514.675777
[K].LLSNLFANYAGADTPVEK.[G]	1922.985818
[K].LTCVVESSVLCAK.[E]	1465.738912
[R].IPVQEEVIEVK.[V]	1282.725294
[K].FGLSKPSEPSEPTITK.[E]	1717.900692
[K].ITGLQEGNTYEFR.[V]	1527.743797

[R].VLDSPSAPVNLNVR.[E]	1480.811818
[K].AVEEVEAPPAAVPK.[R]	1406.752571
[K].TLAFLFTGAAGADAEAGGGK.[K]	1824.912654
[R].EFDDLPLPEQR.[E]	1358.658671
[K].KVPQVSTPTLVEVAR.[K]	1623.942832
[K].TAPSVYPLAPCGR.[D]	1388.699096
[K].EAPFTNFNPSCLFPACR.[D]	2027.910228
[K].NCQALVSDIDYR.[N]	1453.674004
[K].IPLLADVTR.[N]	997.6040568
[R].DSVNLTWTAPASDGGSK.[I]	1705.802769
[R].ELAEAQEDSILK.[-]	1345.684551
[K].NAADSVSEPSESTGPITVK.[D]	1888.913441
[K].TIEEYAICPDLK.[V]	1451.708658
[K].ADAIGLSLIK.[G]	1000.603722
[K].VDKGDTGQYTCYAVNEVGK.[D]	2103.96516
[R].SVSEAGVGESSAPTEPIR.[V]	1772.866097
[R].ADISSFVIESAER.[G]	1423.706349
[K].NYPVVSIEDPFDQDDWK.[T]	2066.934177
[K].EENEVPAPAPAPAPPPGEPSK.[E]	2081.018575
[R].LTAEDLVEAR.[V]	1116.589529
[K].DTGEYTLELK.[N]	1168.57321
[R].YSGAYGASVSEEELK.[R]	1589.732958
[K].LVESGGGLVQPGGSLR.[LV]	1525.833281
[R].DEQDTYAINSYTR.[S]	1575.692156
[K].GCFKPIVAGDQNVEYK.[G]	1824.894895
[K].LIQGNEYLFR.[V]	1252.668448
[R].INLDGLGPDNTVTVIAGNK.[L]	1911.018181
[K].ESHDSASNVYTLELR.[I]	1720.813668
[R].DLSIDDTSQIK.[V]	1234.616137

[R].YGVGPGITSAPVVANYPFK.[V]	1937.016725
[K].NNLPISVSSNVSVSR.[S]	1572.834009
[R].VSNVAGDDACSGILTVK.[E]	1705.842525
[K].IVEGLPINDFSR.[E]	1359.726691
[K].AADSGTYICQLSNDVGIATSK.[A]	2171.028488
[K].LSLEGDHSTPASAYGSVK.[A]	1818.886833
[R].VYSENICGLSDSPGVSK.[N]	1811.848005
[K].VNVDEVGGEALGR.[L]	1314.664819
[K].GLSDAVTIGPITVK.[E]	1370.788957
[K].CEVGTNNWSQCNDTPVK.[F]	2008.84875
[K].SLYNYIQQDTK.[G]	1372.674321
[KR].VNAYNLSDNVYK.[H]	1399.68522
[R].DVVICPDASLEDAKK.[E]	1659.825813
[K].ISNYSYPASPESTELK.[T]	1785.854136
[K].GDTGQYTCYAVNEVGK.[D]	1761.77484
[R].VLQELVNDGPDDRPAGTR.[S]	1951.983193
[R].IGIFGQDEDVTSK.[A]	1408.69545
[K].GNDISSGTVLSDYVGSGPPK.[G]	1949.945076
[R].LIGPNCPGVINPGECK.[I]	1724.845837
[R].SLYSSAENEPPVPLVR.[N]	1757.90684
[K].KGDVTAQVALQPALK.[F]	1538.890068
[R].QLDEKDTLVSQLSR.[G]	1631.85989
[K].FTITDLPTDAK.[I]	1221.636145
[K].GLLPEELTPLVLATQK.[Q]	1722.004763
[K].YDGGQPVTGYLLER.[K]	1567.775098
[K].IPSRPDISSDEDIQYR.[L]	1890.919195
[R].IPVVLPEDEGIYTAFASNIK.[G]	2176.153612
[R].ILTVEGLTEGSTYEFK.[I]	1786.910922
[K].TPVPFGGLLAGETLPR.[A]	1624.905718

[R].APLQVAVLSPTGVAEPVEVR.[D]	2032.143716
[R].SQIDELYSTVK.[V]	1282.652523
[K].DTNAILGGSVVLECR.[V]	1603.810831
[R].SPSWDPFR.[D]	991.4632063
[K].VCNASVTGLSSGQEYQFR.[V]	2002.928715
[K].GPDFLVTEVENGGFLGSK.[K]	1865.927969
[K].TSATLTWEPPLLDGGSK.[I]	1772.906506
[K].SEDYALPVYVDR.[R]	1426.684886
[K].VTNPAGEDVASIFLR.[V]	1588.832947
[K].DVEQTVGLPVTLTCR.[L]	1687.868346
[K].ASASSSGAQVGGSISSGSSASSVTVTR.[S]	2385.148814
[R].SEWGDLLNDLQK.[A]	1417.695785
[R].AGTPSVPELSSGLELLR.[L]	1725.93814
[K].LVTDTPLYVQAVK.[S]	1446.820257
[K].VVDIPDPPVAPNVK.[E]	1459.815506
[R].SNGGSPIQGYIIEK.[R]	1462.753634
[R].TDNLPENLGYHLK.[M]	1513.764533
[K].YSLEPVAVELK.[S]	1247.68818
[K].IDAAEEEKYDMEIK.[V]	1683.778194
[K].KEENEVPAPAPAPAPPPGEPSK.[E]	2209.113538
[R].YAGLKPGELPTCESLK.[D]	1762.904397
[K].LDILDTLDEVK.[V]	1273.688574
[R].VPLQFLPNEAR.[S]	1283.710647
[K].VYVDDGLISLLVK.[Q]	1433.825008
[R].AATESFASDPILYRPVAVALDTK.[G]	2435.281666
[K].LSVVSVEDPPQR.[V]	1325.705956
[R].ISIPGPPETLQVFDVSR.[D]	1854.995989
[R].VDIIHDLPR.[A]	1077.605119
[K].DLQTEASLDSVLCYDPVTAK.[W]	2225.064205

[R].VSAENAAGVGEPSSPTVYYK.[A]	2025.976376
[K].GNNVLGDAIPITAAK.[A]	1453.800918
[R].LGHDFNPDVQAAFQK.[V]	1686.823445
[K].TEAGATLTVK.[E]	990.5466015
[R].LDIQLPVISDDFK.[F]	1502.810086
[K].TLESLVDFSK.[T]	1138.599031
[R].VYGIGSLALYEK.[A]	1312.714729
[R].VSAQNTFGISDPLEVPSVVIIK.[S]	2313.270039
[K].VPQVSTPTLVEVAR.[K]	1495.847869
[R].ASEAVAALDPLDLK.[R]	1412.763136
[R].DADIQNFVSFISK.[D]	1483.742735
[R].LSCAEDYLSLVLNR.[L]	1652.831233
[R].NVNSDTESILASLK.[I]	1490.769678
[K].VNDLVEGVPYYFR.[V]	1570.790019
[K].AENSVGAVASSAVLVIK.[E]	1614.906112
[R].IDALYLPR.[F]	960.551293
[R].LGGEVSCLVAGTK.[C]	1290.672213
[K].CNIPADAPQFIQHR.[V]	1666.811835
[K].AIGIVATTGVSTEIGK.[I]	1516.858099
[K].IQGQGTYQDYLEGLR.[V]	1740.855139
[K].FASDVFSTNFK.[D]	1262.605179
[R].TSVSLAWSVPQDEGGSK.[V]	1747.849719
[K].NSLAHAVQSSR.[H]	1169.602159
[R].ISFIDNIATLQLGSPEASNSGK.[Y]	2262.161216
[K].LVIANALTEDEGDYVFAPDAYSVTLPAK.[V]	2982.49826
[K].LGDGLFLQCCK.[NE]	1310.623155
[R].YTVETDNFSSVLTIK.[N]	1716.869057
[R].VLLFNNAASLGDVSK.[S]	1547.842783
[K].SALAHAVQSSR.[H]	1126.596346

[R].AAAEGSSNVFSMFDQTQIQEFK.[E]	2435.118365
[K].IPVLEEKPAVPVPK.[K]	1515.914492
[R].SVLAQETLGDLR.[V]	1301.705956
[R].AITQDLPGLLGSLGLGK.[N]	1652.958147
[K].VIKPPPVEPPPAPIAAPVTVPVVGK.[K]	2469.484329
[K].VNAEGSVDDVFSQVCTHLDTLK.[-]	2434.155479
[K].ALRPEEEAGIAGDAAPGTAGR.[D]	2009.004656
[R].TEIVSTDYNTLLTVK.[D]	1696.900358
[K].LLCGLLAER.[L]	1044.587027
[R].LADALQDLR.[A]	1014.557835
[K].DTVGVLGTSCILECK.[V]	1651.802969
[R].LATLAALDALAR.[S]	1198.715398
[K].VLQSFSDGLK.[H]	1093.588801
[K].IPGTGALASAAAAVSGSK.[A]	1528.832947
[K].VSEQTPLTALYVANLIK.[E]	1860.04769
[K].ELIIPPEVDLSEIPGAQVTVR.[I]	2275.254389
[K].TVLGAPEVLLGILPGAGGTQR.[L]	2019.1597
[R].ECSAVLTVLEPAR.[I]	1444.74644
[-].APPSVFAEVPQAQPVLVFK.[L]	2024.121524
[K].EDPPACYATVFDK.[F]	1512.667521
[R].VVVAGYTAALNCAVR.[G]	1563.831173
[K].GYDLPVDAISVQLAK.[V]	1588.858099
[K].FDQFFGEGCAPGSQR.[N]	1702.72783
[R].LPAEGPQPAHVVVGDVR.[Q]	1740.939143
[K].VVKPLDVPEGSLER.[D]	1537.858433
[R].SQSVRPGTDVTFICTAK.[S]	1866.937823
[K].FTCITDTPEIVLAK.[N]	1607.834921
[K].FTGSAGIQVVGDDLTVTNPK.[R]	2019.039311
[R].ALVPAAELLESR.[V]	1268.720877

[K].AADFIDQALAQK.[N]	1290.668842
[K].YAASSYLALSASDWK.[S]	1632.790413
[K].YVLTIENGVGEPK.[S]	1418.752571
[R].DDSGVYNISLK.[N]	1210.595008
[K].NNVEIHPTANCEITFK.[N]	1886.906523
[R].ADLTEYVSQHYK.[A]	1453.695785
[K].SRPTSITWDGLNPDK.[L]	1686.844574
[R].APVPASELVAAGVLSQAQFEQLQDGR.[T]	2681.389319
[K].EYLPIGGLAEFCK.[A]	1496.745378
[K].GDVTAQVALQPALK.[F]	1410.795105
[R].GTEYTASGLTTGTEYVFR.[V]	1952.923612
[K].VLDKPGPPAGPLQITGLTAEK.[C]	2102.185581
[K].ALASAAPAGVPLQPQDLEFTR.[L]	2152.139693
[K].NAFVTPGPPGVPEVTK.[I]	1609.858433
[R].LQTLYDALISVADPSK.[TQ]	1733.931992
[K].HDPSLLPWTASYDPGSAK.[T]	1941.934117
[K].AAPAPAPAPAPAPEPPKEPEFDASK.[I]	2453.234716
**lamb**	
[R].VNAYNLSDNIYK.[H]	1413.70087
[RK].AAVQQLQAEGLSPR.[F]	1467.791416
[K].DDPHACYATVFDK.[L]	1538.658019
[K].GTNVLGDAIPITAAK.[A]	1440.80567
[K].LSISETYDLK.[S]	1168.609596
[R].GEAEVGAEALGR.[E]	1158.574941
[R].SPPNPENIAPGYSGPLK.[E]	1737.880625
[K].SLHTLFGDELCK.[V]	1419.693677
[R].VVSALPIQHQDWTGGK.[E]	1735.912594
[K].EISHDPSLKPWTASYDPGSAK.[T]	2286.103702
[R].VTVPNVPIR.[F]	994.6043912

[K].DVIATDKEEIAFK.[D]	1478.773701
[K].LGQEFDEVTPDDR.[K]	1520.686342
[K].VFADYEEYIK.[C]	1276.609596
[R].AVNWAGVGELSAPSK.[L]	1485.769618
[R].AISESGVALTSALVK.[R]	1445.820985
[R].LAAQSGLYESEEL.[-]	1409.679466
[R].SSFFVNGLTLGGQK.[C]	1454.763805
[K].IGKGDAGQYTCYASNVAGK.[D]	1959.922901
[K].VIYQGVNSPGQPVFLEGQQQL.[-]	2301.187372
[K].QDGQFSVLFTK.[C]	1269.647378
[R].LQPPGPEISSYSSQTK.[Q]	1718.859555
[K].LLQDFFDGR.[E]	1110.557835
[R].RANLLQAEIEELR.[A]	1554.85983
[K].DAPSPVDAAFR.[Y]	1145.558563
[K].ECCHGDLLECADDRADLAK.[Y]	2247.942727
[K].GTEDEVEKYSESVKDAQEK.[L]	2170.998627
[K].IVDVLDSIK.[T]	1001.587738
[K].NLEYVATSIHEAVTK.[I]	1674.869726
[R].STHPHFVR.[C]	980.5060742
[K].TPGAMEHELVLHQLR.[C]	1730.900649
[K].NLVHIITHGEEKD.[-]	1504.775432
[R].IQLVEEELDRAQER.[L]	1727.892252
[K].VKLEQHVDDLEGSLEQEKK.[V]	2224.145566
[R].QVTVNDLPVGR.[S]	1197.658611
[K].YSESVKDAQEKLEQAEK.[K]	1981.971291
[R].LQLLEPFDK.[W]	1102.614287
[K].DLPPDTALLDLQNNK.[I]	1666.864641
[K].NADGYIDLEELK.[IL]	1379.668901
[R].DSFPNFLACK.[H]	1198.556121

[R].SVGGSGGGSFGDSLVTR.[S]	1539.739775
[K].VFANPEDCAGFGK.[G]	1411.631076
[K].EGYLQIGVNTR.[EA]	1249.653526
[K].FESLDLAELAK.[K]	1235.651795
[R].IGAEVYHHLK.[G]	1166.631669
[K].KAITDAAMMAEELKK.[E]	1649.86009
[K].LLQDFFNGR.[DE]	1109.573819
[K].VQHELDEAEERADIAESQVNK.[L]	2410.148085
[K].LKGADPEETILNAFK.[V]	1645.879563
[-].SQAEFDKAAEEVK.[H]	1493.711829
[R].CLFGLGEIDDK.[I]	1266.603465
[K].KAEAVATVVAAVDQAR.[V]	1598.886045
[K].ILDDLTIAESNCPR.[G]	1616.794847
[K].TQELLSQLPFK.[EQ]	1303.725628
[R].YWHDTCFR.[C]	1184.494189
[R].ELLLPVLVK.[E]	1023.681245
[K].HGNTVLTALGGILK.[K]	1393.816175
[R].WTEYGLTFTEK.[W]	1374.657608
[R].GIVGSFEELCR.[N]	1266.614698
[R].VLPGILNDTLR.[F]	1210.715398
[R].HVLTTLGER.[M]	1025.573819
[K].GITHIGYTDLPSR.[M]	1429.743404
[R].KDLYANNVMSGGTTMYPGIADR.[M]	2406.106421
[K].IDIDPDETVR.[A]	1172.579358
[K].FVMTIENPAGK.[K]	1206.618721
[K].DIINEQFLLQR.[L]	1388.75324
[R].HGGFKPTDK.[H]	986.5054054
[R].VLPTIDLSTMPQK.[T]	1442.792328
[R].ETNLDSLPLVDTHSK.[R]	1668.843905

[R].VTGLLENHNYEFR.[V]	1591.786331
[K].NKDPLNETVVDLYKK.[S]	1775.95379
[R].LDDECEYACGVEDR.[R]	1730.663241
[K].ELEEIVQPIISK.[L]	1397.788622
[K].SGDRGETGPAGPAGPIGPVGAR.[G]	1975.994426
[K].AGLLGLLEEMRDER.[L]	1601.831567
[K].KLEVNEAELLR.[R]	1313.742341
[R].KVESLQEEIAFLK.[K]	1533.852285
[-R].STPAITLENPDIK.[Y]	1398.747486
[R].LVNHFVEEFK.[R]	1261.657549
[K].LPCIFICENNR.[Y]	1435.682066
[R].SQIDELYSTIK.[V]	1296.668173
[K].YGVGEPLESMAVK.[A]	1379.687528
[R].GKPATLPPPTQVQAFR.[D]	1707.954065
[K-].MLDAEDIVNTPKPDEK.[A]	1814.884056
[R].VLGATLSPELIQK.[V]	1368.809692
[K].DLTLSTRPEEGCSLHEEDAQR.[H]	2443.115405
[K].ESTLHLVXR.[L]	1067.620769
[K].IDLSAIKIEFSK.[QE]	1363.783143
[R].NTDTSVVVTWEEPK.[D]	1604.780243
[R].SWHPEEFNCAYCK.[XTA]	1727.694088
[K].LSVEALNSLTGEFKGK.[Y]	1692.916676
[K].LRLEIPISGEPPPK.[AV]	1545.899904
[K].GYALPPHCSR.[G]	1157.552038
[K].AENEVGIGEPSLPSRPVVAK.[D]	2049.097494
[K].ASSTSPVGISEWLDQK.[L]	1704.843905
[K].DQLIQNLLKEEHVPQNK.[IM]	2046.097828
[K].EIDQEAAVEVSQLR.[D]	1586.802041
[K].GLGTDEDTLNEILASR.[T]	1703.844634

[K].KHADSVAELGEQIDNLQR.[VJI]	2023.020306
[K].KLGLTEEDSVYVFK.[G]	1627.857765
[K].LPEGVTINYK.[S]	1133.620101
[K].YAAELHLVHWNSK.[Y]	1567.801587
[K].YTLPPGVDPTLVSSSLSPEGTLTVEAPLPK.[S]	3065.629274
[R].EAYPGDVFYLHSR.[L]	1553.738318
[R].FASEIAGVDDLGTTGR.[G]	1608.786391
[R].KVQHELDEAEERADIAESQVNK.[L]	2538.243048
[R].LADVEYADVR.[H]	1150.573879
[R].LATALQKLEEAEKAADESER.[GV-]	2202.124831
[R].VDLLSFTGSTQVGK.[Q]	1451.774035
[R].VLFETDLVNPR.[G]	1302.705227
[R].VTAENEYGIGLPAHTDDPVK.[V]	2126.040039
**chicken**	
[K].ACANPANGSVILLENLR.[F]	1811.943243
[K].DDVFLSVPCVLGSSGITDVVK.[M]	2207.126411
[K].DLADELTLVDVVEDK.[L]	1673.847988
[K].DLFDPVIQDR.[H]	1217.616078
[K].VFADYESYVK.[C]	1220.583381
[K].FASELSSLER.[E]	1138.573879
[K].GALVLGSSLQQSGTTR.[K]	1574.849659
[K].LELQAFDQIDDEIK.[L]	1676.837757
[R].IGDEFVADLDQLQR.[L]	1618.807126
[K].LNFEVFPDTTYESTK.[M]	1790.848322
[K].SPPPSSSFFPSNVGK.[Q]	1534.753634
[K].VNVAECGAEALAR.[L]	1359.668524
[R].VAGSQPLTVSWYK.[N]	1435.757991
[K].ATEPVIAFYK.[G]	1138.614287
[K].DVASAQWVSVSSSSK.[K]	1537.749277

[R].VPVPAEIPVTPPTLVWGLK.[N]	2013.178311
[R].LDVPISGEPAPTVTWK.[R]	1709.910863
[K].SFVCSAAPGGALLK.[K]	1377.719497
[K].NEVLIDQTSSALK.[I]	1417.7533
[K].ECQTLVSDVDYR.[H]	1484.668584
[R].EGSNEWVAANTELVER.[C]	1803.850782
[R].IGIFTEDEEVSGR.[A]	1451.701264
[K].LVSWYDNEFGYSNR.[V]	1749.786725
[K].ECQALVSDVDYR.[H]	1454.658019
[K].ISTQDDTSQLLIK.[A]	1461.779514
[K].SSEISEPVFVEASPGTK.[E]	1763.869786
[K].AVNDVGSDSCIGSVTLR.[A]	1749.843588
[K].SSAGAPTYNITATITNK.[S]	1709.870454
[K].KIEEYAICPDLK.[V]	1478.755942
[K-].DSCSFSIDVEAPR.[S]	1482.652934
[R].SAGYGGLGLSVEGPSK.[V]	1478.748549
[K].AISASGTSDPATLEQPVLIR.[E]	2026.08151
[K].NLQQEIADLTEQIAEGGK.[A]	1956.987275
[R].EVNISGITDTEEER.[I]	1591.744585
[K].VADQISDVVYK.[D]	1236.647044
[R].FCGWYDADLSPAGQQEAR.[R]	2070.897414
[K].DYPVVSIEDPFDQDDWEAWK.[R]	2454.077212
[R].ITIETTPNHSTVTITDSK.[R]	1958.007676
[K].QETTPINDNVSQCCSSSYANR.[R]	2431.024876
[K].DFYELDPQK.[F]	1154.536431
[K].FTSVTDAPDVVQAK.[I]	1477.7533
[K].NSVDLTWQPPR.[H]	1312.664425
[K].FITPLQDQTVK.[E]	1289.709978
[K].IEEYAICPDLK.[V]	1350.660979

[K].SSLDGYVVEICK.[D]	1369.666793
[K].LINANFPTNPER.[M]	1385.717189
[K].TLALLFATYGGEAEGGGGK.[-K]	1811.917405
[-].AVTLDESGGGLQTPR.[G]	1500.765261
[K].NLTEEMAVLDETIAK.[L]	1676.841128
[K].STYLTSEPVVAQYPFK.[V]	1829.931992
[K].YVNEFLATALCTQNVNVVEQEK.[I]	2569.260279
[K].IDATAATALASR.[F]	1160.626977
[R].YGAATANYTEVLR.[L]	1428.711769
[K].DKYTTVVDTPENIR.[T]	1650.833341
[K].NLTEEMAALDENIAK.[L]	1661.805077
[R].DPCDPPGRPEAIIIK.[R]	1677.862867
[K].FTISPSTTQVAGILK.[I]	1562.878834
[K].EYLPIAGLADFTR.[A]	1465.768556
[R].GIGWLPNDSPGVQR.[V]	1495.765202
[K].QPAHDAWAEDVDLR.[V]	1622.755759
[-].AASIFAAVPR.[A]	1044.583656
[R].LQEPLGFVK.[V]	1030.593158
[K].YSGGSPILGYYIDK.[R]	1532.763136
[R].VDIPYEAPPDSVLTWLK.[D]	1943.016056
[R].TVPPAVPGVTFLSGGQSEEEASLNLNAINR.[C]	3067.569468
[R].LDEAGGATQAQLEMNK.[K]	1675.795575
[R].EETITYDEWNR.[K]	1455.638664
[K].SELAYEVLDK.[G]	1166.593946
[K].AFEEAAEHFQPYVK.[F]	1665.790748
[R].EAAIIDTTSSFTSLVLDSVNR.[F]	2239.145232
[R].TSDVDSVFFIR.[S]	1285.642293
[K].GVGFGLVDVEK.[D]	1119.604451
[K].TNYHTPPDTFSIQAAK.[K]	1790.870789

[K].GAGEGGLSLAVEGPSK.[A]	1428.732898
[R].DPQYPPGPPAFPK.[V]	1410.705227
[R].FGGEPVGSFVQPR.[L]	1376.695725
[R].IDEFDYSKPLQGQQK.[R]	1795.886104
[K].DCGFKDEGEYTVTAGQDK.[S]	2019.860026
[R].ETTTTTWDVVSAAVAR.[T]	1707.854804
[K].VSDNLETAVNLAWTAGSNSTR.[F]	2206.073464
[K].YTPSGHAGAAASESLFISNHAY.[-]	2251.041436
[K].YDFAEQIK.[R]	1013.493838
[R].GLCFSAENEPVCR.[M]	1538.672624
[R].IAELLGLDK.[N]	971.5771734
[R].VYGTGALALYEK.[A]	1284.683429
[R].LPLDVAYR.[R]	946.535643
[K].CFTEVFVK.[E]	1029.50738
[R].ASDAGPVFTPPVK.[D]	1285.678678
[K].AALEALDELDLFGAK.[G]	1575.826464
[R].TSCTISDLIIGNTYSFR.[V]	1947.948053
[R].VTVVQEADTVEICGALK.[N]	1831.94699
[R].EPADAMAAGAVEASFK.[C]	1564.731184
[R].FDAIPIQAAK.[A]	1073.598971
[K].SSPLDSKPVVVQYPYK.[V]	1806.963627
[K].ITVTEAEDAFINK.[E]	1450.742401
[R].LAVFAQPIVSK.[A]	1172.703771
[R].IIYGGSVTGGNCK.[E]	1325.651812
[K].FNITAIPK.[G]	903.5298293
[R].DVSTEFTVDAR.[A]	1239.585172
[K].TIALAVPLAQK.[T]	1124.703771
[K].SPFPVTVAPPLQLDK.[V]	1608.89957
[R].DGFNLLLPK.[L]	1016.577508

[R].GAGSGALGLTVEGPR.[E]	1341.712104
[K].LADNLDTLSAAAAK.[L]	1373.727085
[K].VTEATITGLIQGEEYTFR.[V]	2028.028411
[K].AIADYNVLPATENPLLR.[Q]	1870.006888
[K].ATGGTEITGYYVNYR.[E]	1664.791476
[K].VISDLNVGPDATR.[V]	1356.711769
[K].IVVGPSEIGNAER.[V]	1340.716855
[K].VENTVGEATSSSLLTVQER.[K]	2020.019303
[K].ATGYLLPPDTVQIR.[H]	1543.847869
[R].GLPGESGAVGPAGPIGSR.[G]	1578.823445
[R].GDPGPVGPVGPAGAFGPR.[G]	1604.817966
[K].EGPYDVIVLPGGNLGAQNLSESAAVK.[D]	2598.340972
[K].DLETVMFDR.[S]	1125.524486
[K].ALQEAHQQTLDDLQVEEDKVNTLTK.[A]	2866.44287
[K].FFSASCVPGATIEQK.[L]	1641.794119
[R].GVGWLPIQSLEVEK.[A]	1554.85262
[R].ILNVEVADVGEYQCK.[A]	1736.852362
[R].QLLFTADNR.[V]	1077.568734
[K].DIEHPVGLPLK.[L]	1217.688849
[K].MTEEEVEELMK.[G]	1367.606895
[MK].DGLPAGAGVGGAGLAGAGAVGSPYGK.[D]	2127.082907
[K].NTAGAISPPSESTGTIICK.[D]	1903.942968
[K].DYTPEVHAAFDK.[F]	1392.643021
[R].VTGLFEGNTYEFR.[V]	1532.737984
[K].GYDLTPEAISVK.[A]	1292.673258
[R].VGQIATFAVDCR.[D]	1336.667796
[R].VIAQNDIGESEASPASEPVVCK.[D]	2300.107467
[K].GQTVSQVHNSVGALAK.[A]	1595.849994
[R].IALGDDSPAIAQK.[R]	1298.695056

[R].DIENQTVLTDEDAIFECEIK.[I]	2382.101712
[K].SSVTLTWEPPLIDGGSK.[I]	1786.922156
[K].YTVSPLTEGSLYVFR.[V]	1731.895213
[K].LVIPSVTEANSGR.[Y]	1342.732505
[R].VAGAALPCAPAVK.[W]	1224.676904
[K].LETDIVQIQSEMEDTIQEAR.[N]	2348.128596
[R].VAAENAIGQSDYCEIEDSVLAK.[D]	2382.112946
[K].GPEWHTGLESEPGPTVK.[E]	1820.881353
[R].LFFVSENPADQNEK.[H]	1637.780577
[K].FLSAVSAVLAEK.[Y]	1234.704165
[R].LNEFNPCSVYSTK.[S]	1558.72062
[R].SNTPILVDGKDVVPEVNK.[V]	1924.038582
[K].DLYANNVLSGGTTMYPGIADR.[M]	2228.065208
[K].LESDISQIQSEMEDTIQEAR.[N]	2322.07656
[R].GIGWSPLGSLESEK.[N]	1459.742735
**duck**	
[K].DQATYTISLSNQR.[G]	1496.733961
[K].TAEATPIPILLPLVPTPEEK.[K]	2129.210399
[K].AVNDVGSDSCVGSVTLR.[A]	1735.827938
[K].ATEPVITFYK.[G]	1168.624852
[R].VYELQSDNLYK.[S]	1371.679072
[K].ECQTLVSDIDYR.[HQ]	1498.684234
[R].ATDVGPVFTPPVK.[D]	1327.725628
[K].SPPPSSSFLPSNVGK.[Q]	1500.769284
[K].AQNIIGLSLPDTTIPIECQEK.[L]	2340.211538
[K].WSQTPCYDVAVAK.[M]	1524.71514
[K].DVASAQWVPISSSSK.[K]	1561.785662
[K].LVIPTVTEANSGR.[Y]	1356.748155
[R].VPLILGSPDDVK.[E]	1252.714729

[R].IIQELEPTTVESGKPAR.[F]	1868.012368
[K].NTVDLSWQPPR.[H]	1312.664425
[R].LSQTEPITLVR.[D]	1256.720877
[K].EGVECEVINLR.[T]	1317.646726
[K].ACANPATGSIILLENLR.[F]	1812.963644
[K].LAILENANVLAR.[Y]	1296.763411
[K].ECQALVSDIDYR.[H]	1468.673669
[R].NAPYSGYSGAFQCLK.[D]	1662.758068
[R].VLDTPGPVADFK.[A]	1258.667779
[K].LELQAFDQIDEEIK.[L]	1690.853407
[R].APVDPPGKPEVIDVTK.[S]	1661.910863
[K].LVSWYDNEYGYSNR.[V]	1765.781639
[K].IGVSEPSDISEPQVAK.[E]	1655.848656
[R].VAGSQPLTAAWYK.[N]	1391.731776
[K].GQTVQQVQNSVGALAK.[A]	1627.876209
[K].FISPLQDQTVK.[E]	1275.694328
[K].ELAEASEDSILK.[-]	1304.658002
[RK].DLADELALVDVVEDK.[L]	1643.837423
[R].IYGTGALALYEK.[A]	1298.699079
[R].NAAGIFSEPSESSGAITAR.[D]	1864.903545
[K].EDVFLSVPCVLGNNGITDVVK.[M]	2275.163859
[K].FFSASCVPGATTEQK.[L]	1629.757733
[K].VLDTPGPPQNLVVK.[D]	1476.842055
[R].VEDSGDYSCEAQNPAGSASTSTSLK.[V]	2561.094395
[K].FGQSQPSEPTEPIITK.[E]	1758.890856
[R].ACDILYPPGPPSNPK.[V]	1625.799204
[R].QETSPINDNVSHCCSDSYAYR.[R]	2503.024876
[R].YGIGPAITSESIVANYPFK.[V]	2027.048419
[K].LLTFTTTNPPSK.[K]	1319.720543

[K].FTGSVGIQVVGDDLTVTNPK.[R]	2047.070611
[R].VPVPTETPATPPTLVWGLK.[N]	2003.12119
[K].SFVSDANCILK.[F]	1253.619449
[R].ALGVPVIAEDLTIAPSFK.[L]	1841.041877
[K].FEEPNFEETPK.[-]	1366.616137
[K].QVIGSGSFFPK.[G]	1166.620435
[R].SLSQCDLVQNIK.[D]	1404.71514
[R].VLDTPSAPINLTIR.[E]	1509.863519
[R].VNAYNISDNYYK.[T]	1463.680135
[K].DTGEYLLTVSNSAGSK.[T]	1641.796621
[R].IVESLQSSLDAEIR.[S]	1559.827527
[K].EYLPIGGLADFTR.[A]	1451.752906
[K].FGTGPPVEIGPILAVDPLGPPTAPER.[F]	2597.397364
[K].EALDNFSNYTSVVDTPDIVLAK.[I]	2411.197661
[K].NEAGEDTALINIK.[V]	1387.706349
[K].SDGGTPITGYVLER.[R]	1464.732898
[K].YVPPPFNPDVFNFDEK.[L]	1924.911591
[R].DFTPGGIGGALR.[R]	1160.605848
[K].SDPNQHLQIVQDPEYR.[R]	1938.930429
[K].AGEDVQITIPFK.[G]	1317.704893
[K].IADFSTVLINK.[D]	1220.688515
[K].GNNVSLDAIPIAR.[A]	1339.732839
[R].ADIAALADEFK.[Q]	1163.59428
[R].TLNEVVIASAAR.[T]	1243.700476
[R].VYAENAAGLSLPSQNTPLIR.[A]	2114.124043
[K].FTSITDAPDVVQAK.[I]	1491.76895
[K].DILSPPEVNLDVTCR.[D]	1727.863261
[R].FFEAVYPTEAR.[K]	1329.647378
[R].DSVNLTWNEPATDGGSR.[I]	1818.825295

[K].NPFVVPGPPK.[A]	1051.593492
[R].GEVLCTVTTAQPLDDASLTDLK.[S]	2347.169732
[K].ASAFALQDQPVVNAVIDDTTK.[E]	2203.124103
[R].QSEEAEELSNVNLSK.[F]	1676.797349
[K].STGLTDGIAYEFR.[V]	1429.695785
[K].ANADYNVLPATENPLLR.[Q]	1870.965752
[R].LEIPITGEPTPK.[V]	1294.725294
[K].GINTLVGYDLVPEPK.[I]	1614.873749
[R].VDSGQYILLAK.[N]	1206.672865
[K].YTLTPDVPQFIQAR.[Y]	1648.869332
[K].LVGFQSLEDDPK.[L]	1347.679072
[R].SSDPAPTAVPQSDVYR.[M]	1689.807854
[K].DGGSPILGYIVECQK.[T]	1635.804683
[K].QGFNVVVESGAGEASK.[F]	1578.775826
[R].VVFDDSFDR.[S]	1099.505465
[K].DAIAQFEASAVGK.[Q]	1306.663756
[R].LSTGVEYQFR.[V]	1199.605513
[K].TIALALPVAQK.[S]	1124.703771
[K].IETDNYSTVLTIK.[D]	1496.784265
[R].LGSESYSLGLK.[D]	1153.60993
[R].IAPLAEGALPYNLAELQK.[Q]	1911.05859
[R].DEGFNVTAGVDR.[E]	1279.59132
[K].AEDFSLPAYVDR.[R]	1382.658671
[R].AALQQALADLK.[G]	1141.657549
[R].APAFQSISPVSR.[V]	1259.674262
[K].TLEEEVTVIK.[G]	1160.640896
[K].YLLPPDAPELVNAIK.[N]	1652.925785
[K].SLPSIFLDEICQVEK.[L]	1777.904063
[R].VSGVPKPTLTWEK.[D]	1441.804941

[R].TPFGTYGGLLK.[GD]	1153.625186
[K].LPPNVVAEPDLLK.[A]	1404.809692
[K].LSPESEDLEITK.[L]	1360.684217
[K].NLTEEMAALDETIVK.[L]	1676.841128
[K].VTVQTEAGETLTVK.[E]	1475.795164
[K].GLEATLECEVTGTPPFEVK.[W]	2077.015798
[K].FNPETDYLTGTDGK.[K]	1557.706743
[R].SLIGQYTCTATNAIGTASSSGR.[L]	2216.061185
[K].WVTDDIEIK.[T]	1118.572816
[K].TIQLQPTVVER.[G]	1283.731776
[K].VLAQPEQASSAPVLR.[S]	1565.864581
[K].LDVAPISDIIEIK.[S]	1425.819923
[K].GCFKPIIAGDQNVEYK.[K]	1838.910545
[K].SELAYEVLEK.[G]	1180.609596
[R].GVGWLPIQSLDVEK.[A]	1540.83697
[K].VSGTPELSTGWFK.[D]	1408.710707
[R].DLPDLCYVAK.[E]	1193.587086
[R].VSAENAAGVGEPSQASPYFK.[A]	2008.96106
[K].AYDLQSDVVYK.[S]	1300.641958
[K].TQVHLPVDALSVQAAK.[E]	1676.932995
[R].TDEGQPWVLPVVR.[K]	1495.790354
[R].AQNDAGYGTPTELTIVAR.[D]	1876.939931
[K].LENLETTADLAIEAEPIQFTK.[S]	2346.207498
[R].QVILGGDAVTVAR.[S]	1298.742675
[R].VNAESSENITVLTIK.[E]	1617.869392
[R].SPPHPDNIAPGYSGPLK.[E]	1746.88096
[K].DIQETVGAPVTFDCR.[I]	1707.800661
[K].NALTIDENTIER.[I]	1388.701598
[R].TEMNHTGQYTCTATNAVGTATSSAK.[L]	2602.150805

[R].LSTIDEVISTSPLYQTVPDFQR.[V]	2509.28206
[R].IEDQQVIEAQLQK.[K]	1541.816962
[K].MTEEEVDELMK.[G]	1353.591245
[K].VWADIPAPK.[R]	996.551293
[R].WEVSDLQPQLK.[V]	1342.700142
[K].TQVSVPSDTPVMLQSK.[V]	1716.883662
[K].NLPDLEVNENDTVK.[L]	1599.786056
[K].DDTSSVLELFSAR.[M]	1439.701264
[R].VALSPAGVQALIK.[Q]	1266.777998
[K].LLCDLISK.[H]	961.5386797
[K].GQNVEQVVYAVGALAK.[A]	1645.890796
[R].LGGEVSCLVAGTSCDK.[IV]	1652.761833
[K].VVIGTQEPQVLR.[K]	1338.773976
[R].YTPGIVSAFGK.[I]	1139.609536
[R].SLGGSGGGSGLGENLVTR.[S]	1617.819088
[R].SPCVASCNIPVVTGK.[E]	1588.782174
[K].SSAALTVLEEDLR.[I]	1403.737649
[R].QFEEAEQQANSNLVK.[Y]	1734.829318
[K].AVDSQILAQIR.[K]	1213.689912
[K].IELLGTYDPQK.[Q]	1276.678344
[R].MAEILSGVEPVPLTQTAQEALR.[E]	2353.243172
[R].VNVSDSTDLTILNIK.[E]	1631.885042
[K].VSNVAGSVTCSANLFVK.[E]	1752.894895
[K].IEDEQLLGMQLQK.[K]	1544.79887
[R].VTNDVGSCLCSSEVTLK.[E]	1868.87284
[R].FQDENFILK.[H]	1153.588801
[K].LITQLWEK.[V]	1030.593158
[R].IIYGGSVTGSNCK.[E]	1355.662376
[R].GPYFLTADTLK.[S]	1225.646315

[K].DFTATDLTEHAAR.[A]	1447.681197
[K].GTDPEETILNAFK.[I]	1434.7111
[K].GANSVVCQVTHDGTPIEK.[T]	1911.922901
[K].AVSIDVTEGDPATLQCK.[F]	1803.879305
[K].IDELSLYSSPAR.[E]	1350.689971
[R].IPQVSAADSGEYVCR.[V]	1651.774446
[K].VALDTVFVPNTGK.[K]	1360.747092
[R].GTCLVVPDTPQILLAK.[N]	1724.961518
[K].SDSAITWNNLQGK.[K]	1433.701933
[R].GWSIVSSDITK.[R]	1192.620829
[R].GTDKWETCGEPVIETK.[M]	1849.863655
[K].DLQIELDDTQR.[Q]	1345.659399
[K].VEFTPDQIEEFK.[E]	1481.715851
[R].FISLLDELQK.[T]	1205.677616
[K].SVSLDNLSVDEVAK.[A]	1475.758779
[R].DLEEATLQHESTAAALR.[K]	1854.919195
[K].LGEEFDETTADDR.[KH]	1497.633972
[K].YTLDADVPQFIQAR.[V]	1636.832947
[R].LECQISAIPTPR.[I]	1384.725311
[K].LLTPIETLTVDQIR.[Q]	1611.931598
[R].VGVPVPVPALTVNR.[L]	1417.85256
[K].GQTVEQVHNAVGALAK.[A]	1621.865644
[K].EYTITPEADLSDIPGGQIAVR.[I]	2245.134667
[K].GLCDELALVDVLEDK.[L]	1688.841128
[K].IFTVEGSLCSGK.[G]	1297.645664
[K].NLQEEIDALESR.[V]	1416.696513
[R].TEESLDSVLCYDPVAIK.[W]	1938.936485
[R].APVPAPAPASAVGAPAPK.[Q]	1568.879503
[K].VLDTPGPPVNLIVK.[E]	1461.867542

[K].VTDQLSDVVYK.[D]	1266.657608
[K].GGVNECSAYLFVR.[D]	1471.699825
[R].NNLLLAEVEELR.[A]	1412.774369
[R].DAGLQLQAYR.[Y]	1134.590198
[K].LLDAAISCAEK.[I]	1190.60855
[R].ILDDPSPPQPGEER.[L]	1549.749277
[R].ITAEDLYEAK.[I]	1152.578295
[K].SLSLGPALIYSAK.[F]	1319.756928
[R].DVMLEELSLLTNK.[G]	1504.792722
[R].AEGAASSNVFSMFDQSQIQEFK.[E]	2421.102715
[R].NITYLPSGQSVLLQLPQ.[-]	1871.027289
[K].VIFPAPPPNNIPQGAPVK.[Q]	1856.04288
[K].VENSVGEAASSSLLTVQER.[K]	1975.993089
[K].DFNMPLSISR.[L]	1179.582669
[K].FISPALTCIEK.[Q]	1278.676236
[R].IVAFADAAVDPIDFPIAPAYAVPK.[I]	2471.322074
[K].DLLASVIGPEK.[S]	1141.646315
[K].LEAPDADELPK.[L]	1197.599759
[K].IPMAVTAENLAAK.[Y]	1328.724248
[K].TIIAQNPAAEPLK.[N]	1365.773641
[K].YGISFIDGLATLK.[V]	1397.767493
[R].KLAEQELLEATER.[V]	1529.816962
[K].MGLVDQLVDPLGPGVK.[T]	1637.893104
[K].WAVIGDENYGEGSSR.[E]	1639.734689
[R].MATQASTLYSNNIIK.[L]	1654.846882
[K].QQLLADPLVPPQLTIK.[D]	1774.047297
[K].LEQQVDDLESSLEQEK.[K]	1889.897457
[K].NAAGLFSQPSETTGPVTVK.[D]	1903.975982
[R].AETYPDSSCLVIDTAER.[E]	1926.874948

[K].EGNICTLSCQFSIPNAK.[S]	1938.904808
[R].VLAVNEYGVGLPAETPDPIK.[V]	2082.111747
